# Medical Report of the Neilgherry Mountains

**Published:** 1823-04

**Authors:** R. Orton

**Affiliations:** Assistant Surgeon 34th Regt.


### Art. VIII.—*Medical Report of the Neilgherry Mountains.* By R. Orton, Esq. Assistant Surgeon 34th Regt.

[Concluded from page 215.]

A considerable number of European gentlemen have already visited the Neilgherry, for the recovery of their health; and the results of their experiments seem, in general, to have been favourable. Mr. Sullivan, collector of Coimbatoor, attributes a complete renovation of his health, which had long been in a bad state, to a residence on these mountains, after having tried a voyage to the Cape of Good Hope in vain. He has, probably, paid more attention to the subject than any other person, and has formed a very high opinion of the salubrity of the climate. He states, in a letter to me, " M. Letchtenault, French naturalist, came to me at Coimbatoor, in November 1818, with fever; he was confined to his bed, and in a very dangerous state, until January, when he proceeded to Pondicherry. He returned in April, in a most miserable state of weakness and suffering, hardly able to walk, without appetite, and with his skin the colour of saffron. He accompanied me up the hills in May; and I never saw so great and so rapid a change in any man. He was able to take constant walking exercise; his appetite and strength were completely restored in less than three weeks, so that he was able to go from stage to stage on foot. Mr. Jones, the assistant surgeon here, was one of the party: he had been a fever subject for four years, and in the months of February, March, and April, the attacks were repeated and violent. The hill-air completely set him up, and he has had no fever since."

The military chaplain of Trichnopoly visited the Neilgherry, in consequence of a severe chronic disease, (of what nature I am not informed,) but was shortly afterwards attacked with dysentery, and obliged to return for medical assistance. Captain Dun, deputy quarter-master general of the southern division, made trial of the climate on account of severe head-aches, arising from a fracture of the skull; and derived very great benefit from it. When I first saw him, on his arrival there, he was very sallow and thin, and suffering greatly from his local affection; but when he left the place, about four months afterwards, he had fully recovered his flesh, strength, and looks, and almost entirely got rid of his head-ache.

Mr. Campbell, of the 34th regiment, who went up along with me on leave of absence, had been slightly unwell all the way thither, and, some weeks after his arrival, had a very severe attack of remittent fever, with affection of the liver; but he had a very rapid recovery, and left the hills in high health.

Assistant-surgeon Stoddard, of the Royal Scots, went thither in consequence of violent palpitations of the heart, severe intermittent head-aches, and other nervous affections; and in a short time his health was restored to its usual state: but his illness appeared to be owing to excessive mental labour, as he had the whole duty of his corps to perform in a very hot season, at Trichinopoly, and probably would have been removed in any climate, on his being relieved of so intolerable a burthen.

Lieutenant Cozens, of the 25th N. I., went to the Neilgherry, labouring under epilepsy and chronic dysentery of long standing, for which he had been repeatedly advised to return to Europe. In the course of a month he found himself much better. He subsequently relapsed; but, upon the whole, expressed himself much benefited by the climate when I left him. He appears to be a very proper subject for the experiment. If the climate should restore his health to any tolerable condition, it will be a strong argument in its favour. It is probable that it is in such cases as this, in chronic hepatitis and various other local chronic affections, and in general relaxation and debility of the constitution arising from heat of climate, that the climate of the Neilgherry will be found most beneficial. In the present state of our experience on the subject, it certainly does not appear advisable for fever subjects to venture upon the experiment.

I was induced to make trial of the climate chiefly for an affection which appeared at first to be only hernia humoralis; but subsequently, from its long standing and other symptoms, had assumed the more alarming form of sarcocele. My general health was also completely deranged. I was much emaciated and debilitated, and incapable of digesting a sufficient quantity of food. Since suffering severely from Seringapatam fever, in 1815-16, I had always been a valetudinarian. On my way to the Neilgherry, I had one paroxysm of fever at Bangalore, and many more during the first half of my residence on the mountains, which appeared to prevent the climate having any considerable good effect, either on my constitution or the local affection. Subsequently, however, I recovered rapidly in every respect. I had another return of fever just before my leaving the Neilgherry, which proved rather obstinate, but did not materially throw me back; and I have since enjoyed a much greater share of general health, flesh, and strength, than has fallen to my lot for years back, though the local disease is not yet entirely removed, nor free from danger of relapse.

I noticed a striking peculiarity in the intermittent fever of the Neilgherry, in almost all the cases which came under my observation: that is, the absence of the sweating stage. In all the attacks which I experienced, there was scarcely ever the smallest appearance of unusual moisture on the skin. I found, also, that very small quantities of mercury quickly affected the mouth. This was probably owing to the cold preventing its passing off by the skin. It seems to show that the very free use of calomel, which we are in the habit of making in this country, would be improper in the temperate climate of the Neilgherry, as it is in England. It is evident that a very remarkable suppression of the perspiration takes place, in consequence of the sudden transition to the cold of Neilgherry, from the great flow of urine which universally occurs for some time after entering the climate. May not this suppression be one of the causes of the attacks of fever, which appear at the same time? The rigors are also very trifling in this fever. Mr. M'Pherson, the officer of pioneers stationed there, informed me that enlargement of the spleen was a very common attendant on the fever which his party suffered from so severely at Seraloo; and that those who died were generally carried off by a chronic dysentery, which supervened to the fever.

The temperature of the climate is already well known, from registers which have appeared in public prints, and which appear to have been carefully kept by Mr. M'Pherson. I subjoin a table of the monthly extremes for twelve months at Jackanary, compared with those of eight months of the preceding year at Madras; the former being extracted from Mr. M'Pherson's registers, the latter from those kept at the Military Male Asylum. From this very imperfect comparison, it appears that the mean difference is about 18°. This however relates only to Jackanary, which is about 5000 feet high. The Todiernaud is nearly one-half higher, and may therefore be supposed to be about 25° colder than Madras. I observed, by the thermometer, the Todiernaud to be about 8° colder than Dimhutty. The most western part of the range, about the remarkable mountain called Moocarty, is probably much colder than even Wuttacamund; at least, the Toders of the latter place informed us so; and Mr. M'Pherson, during an excursion which he made into that part of the hills, found the thermometer sink as low as $29\frac{1}{4}$—$24\frac{1}{4}$, on the 25th and 26th February. The western extremity has been very little explored by Europeans.

I have endeavoured to ascertain the height of the Neilgherry by means of the boiling point of water, and annex the result of these observations in a table. It cannot be considered as any more than an approximation to the truth; but I found the calculation to agree, within one hundred feet, with one made with the barometer, by a scientific gentleman at Dimhutty; the mercury being found to stand there at 23-9.

In the event of government forming an establishment for invalids on the Neilgherry, it will require mature consideration to fix on the most proper situation for that purpose. The neighbourhood of Dimhutty would be most convenient for procuring supplies, &c. by the new road, and on account of the collector's establishment being placed there; but the country about Cadavamoody is much prettier, more cultivated, and populous. The Todiernaud, from its much greater height and coldness, would deserve the preference, were it not to be apprehended that its swamps and woods would be injurious. It does not appear, from experience, that such is the case; for the Toders are a more robust and healthy race than the other casts, who occupy the less elevated parts. It is probable that a clear and dry tract may be found, equally elevated with the Todiernaud. The country immediately west of Soloor seems to possess all these advantages; and it is close to Mysore, and a pass leading down into it. It might be deemed advisable to erect a few small temporary buildings for the reception of sick officers, similar to some already raised by Mr. Sullivan for travellers, at several different parts of the table-land. The experiment of proving, on a sufficiently large scale, the effect of this singular climate on the European constitution, would be a highly interesting one, in a philosophical point of view; and, if successful, its results would be extremely important and valuable.

**TABLE,**

*Showing the Temperature of the Neilgherry in comparison with that of Madras.*

| | *Mean Heat in a House at Sunrise.* | | *Mean Heat in a House at Noon.* | | *Mean Differ. between Neilgherry and Madras.* |
|---|---|---|---|---|---|
| | Neilgherry. | Madras. | Neilgherry. | Madras. | |
| March | 58¾ | 76½ | 70 | 85¼ | 16½ |
| April | 60¼ | 83 | 70¼ | 89 | 21¼ |
| May | 60 | 82½ | 67½ | 90½ | 22¾ |
| June | 59½ | 85¼ | 72½ | 90¾ | 22 |
| July | 58¾ | 80¾ | 66¾ | 87¾ | 21½ |
| August | 58½ | 80¾ | 67 | 86 | 20¾ |
| September | 58½ | 80¾ | 65½ | 88 | 22½ |
| October | 57 | 79¾ | 65½ | 86¾ | 22 |
| November | 54½ | 77¼ | 63¼ | 84 | 21¾ |
| December | 55½ | 75½ | 64½ | 81¼ | 18½ |
| January | 51¼ | 75 | 63 | 82¾ | 21½ |
| February | 49 | 74¼ | 63 | 84½ | 23¼ |
| Annual Means | 56¾ | 79¼ | 65½ | 86¼ | 21½ |

*Remarks.*—The above table is extracted from Lieutenant M'Pherson's registers, kept at Jackanary, and those of Mr. Goldingham, astronomer to government, kept at the Madras observatory; the means of *all* the observations for the period being taken. Hence it appears that the mean temperature of the former place is 21½° lower than that of the latter. The Todiernaud is about 2000 feet higher than Jackanary, and, if the decrement of temperature continues in the same proportion,—that is, about four degrees and a small fraction to the thousand feet of elevation,—that tract will be about 30° colder than Madras.

It is probable that this will be found very near or within the truth; for it appears from Mr. M'Pherson's registers, that the mean temperature of Seroloo is about 9° higher than that of Jackanary; the former place being about 2000 feet lower than Jackanary. It may be remarked, too, that all these results agree pretty nearly with the theory laid down in Dr. Black's Lectures on Chemistry, of one degree of diminished temperature to every two hundred feet of elevation.

TABLE *of* EXPERIMENTS *on the* ALTITUDE *of the* NEILGHERRY MOUNTAINS, *&c. by means of the Boiling Point of Water.*

| | *Boiling Point found.* | *Probable Elevation above the Sea, at the rough Calculation of 555 Feet to each Degree of the Boiling Point below 212.* |
|---|---|---|
| Naickanary, at the top of Pednadroog Pass | 209 | 1665 Feet |
| Colar in Mysore | 208 | 2220 |
| Bangalore | 207½ | 2497 |
| Sreemoka in Coimbatoor, at the foot of the Neilgherry mountains | 210½ | 832 |
| Seroloo, on the edge of the Neilgherry | 206 | 3330 |
| Jackanary | 202½ | 5275 |
| Dimhutty | 201½ | 5827 |
| Codavomoody | 202 | 5550 |
| A high peak close to Codavomoody | 200 | 6660 |
| Denaar | 202 | 5550 |
| Soloor | 202 | 5550 |
| Wattacamund | 199 | 7215 |
| North peak of Bewy-bet | 197 | 8315 |

